# The Complement System Is Critical in Maintaining Retinal Integrity during Aging

**DOI:** 10.3389/fnagi.2018.00015

**Published:** 2018-02-15

**Authors:** Ryo Mukai, Yoko Okunuki, Deeba Husain, Clifford B. Kim, John D. Lambris, Kip M. Connor

**Affiliations:** ^1^Angiogenesis Laboratory, Department of Ophthalmology, Massachusetts Eye and Ear Infirmary, Harvard Medical School, Harvard University, Boston, MA, United States; ^2^Department Ophthalmology, Graduate School of Medicine, Gunma University, Maebashi, Japan; ^3^Department of Pathology and Laboratory Medicine, University of Pennsylvania, Philadelphia, PA, United States

**Keywords:** aging, complement system, retina, ERG, OCT, EM

## Abstract

The complement system is a key component of innate immunity comprised of soluble components that form a proteolytic cascade leading to the generation of effector molecules involved in cellular clearance. This system is highly activated not only under general inflammatory conditions such as infections, collagen diseases, nephritis, and liver diseases, but also in focal ocular diseases. However, little is known about the role of the complement system in retinal homeostasis during aging. Using young (6-week-old) and adult (6-month-old) mice in wild type (C57BL/6) and complement knockout strains (*C1q*^−/−^, *Mbl a/c*^−/−^, *Fb*^−/−^, *C3*^−/−^, and *C5*^−/−^), we compared amplitudes of electroretinograms (ERG) and thicknesses of retinal layers in spectral domain optical coherence tomography between young and adult mice. The ERG amplitudes in adult mice were significantly decreased (*p* < 0.001, *p* < 0.0001) compared to that of young mice in all complement knockout strains, and there were significant decreases in the inner nuclear layer (INL) thickness in adult mice compared to young mice in all complement knockout strains (*p* < 0.0001). There were no significant differences in ERG amplitude or thickness of the INL between young and adult control mice. These data suggest that the complement system plays an important role in maintaining normal retinal integrity over time.

## Introduction

Retinal degeneration is a common form of neurodegenerative disease and a leading cause of vision loss worldwide. In the United States alone, about 3.4 million people are estimated to have vision loss due to retinal degeneration, and its prevalence is predicted to increase in industrialized countries with aging populations (Wert et al., 2014). Retinal degeneration contributes to vision loss in many conditions, including age-related macular degeneration, retinal detachment, and retinitis pigmentosa. There is considerable genotypic and phenotypic variability with retinal degeneration which complicates the identification of mechanisms of degeneration and development of potential therapeutic approaches for this debilitating condition (Wert et al., 2014). The complement system has recently become a topic of intense investigation, and is both a key mediator of developmental neuronal function and homeostasis, and contributes to a number of retinal disease pathologies (Sweigard et al., 2014, 2015; Kim et al., 2016). Cumulatively, these seminal studies suggest that the complement system is vital to normal retinal physiology.

The complement system is an essential component of the innate immune system and plays an important role in immune function and disease pathogenesis. The complement system is a hub-like surveillance network of the innate immune system that plays a vital role in the modulation of immune and inflammatory responses (Walport, 2001a,b; Harboe and Mollnes, 2008; Ricklin et al., 2010; Yanai et al., 2012). Activation of the complement system involves the sequential cleavage of proteins that resembles the coagulation cascade (Barnum, 2017). There are a number of proteins that comprise the complement system's three main upstream activation pathways—classical (complement components C1–C4), alternative (Factors B and D), and lectin (MASP-1 to 3 and MBL)—as well as the terminal activation pathway (complement components C5–C9). Additionally, the system is regulated by soluble (e.g., Factor H, I) and membrane-bound (e.g., CD55, CD59) complement regulatory proteins (Barnum, 2017). While liver hepatocytes are the primary source of complement synthesis, several other tissues and cell types, ranging from macrophages to endothelial cells to neurons, are capable of complement synthesis when induced, and may synthesize some components constitutively under certain circumstances (Barnum, 2017). Based on both synthesis location and function, it is evident that the complement system can affect all parts of the body, so it is an important consideration in various focal and systemic pathologies.

The complement system is complex, with numerous complement effectors and a variety of receptors with different effects (McGeer et al., 2017). Thus, any dysfunction or defect in this complex network will likely be of consequence, as evidenced by the identification of complement involvement in numerous systemic (McGeer et al., 2017), inflammatory (Zhuang and Lyga, 2014), and retinal (Zhuang and Lyga, 2014; McHarg et al., 2015; Xu and Chen, 2016) pathologies. Indeed, the complement system has recently been implicated in a variety of pathophysiological processes, including photoreceptor degeneration, vascular regression, ischemia/reperfusion injury, sepsis, stroke, autoimmunity, and inflammatory disorders (Guo et al., 2005; Walsh et al., 2005; Sjoberg et al., 2009; Langer et al., 2010; Ricklin et al., 2010; Sweigard et al., 2014, 2015). Unsurprisingly, there is considerable interest in the development of complement-based therapeutics, with numerous clinical trials conducted and underway (Melis et al., 2015; Ricklin and Lambris, 2016).

The complement system is thought to contribute to general homeostasis by eliminating immune complexes and apoptotic cells, as well as mediating cross-talk with immune cells for adaptive immune functions (Ricklin et al., 2010). During synapse development and plasticity, C1q, the initiating protein of the classical complement pathway, is believed to play an important role in synapse elimination (Stevens et al., 2007). Moreover, C1q protein levels dramatically increase during normal aging in mice and the human brain (Stephan et al., 2013). In the study of sleep and circadian rhythms, the plasma level of complement components C3, C4, C3a, and C5a appear to fluctuate with the sleep-awake cycle, thereby suggesting that circadian rhythms can affect the immune regulatory properties of the complement system (Reis et al., 2011). In retinal detachment models in mice, deficiencies in key complement components (i.e., Factor b, or C3) resulted in a decreased number of apoptotic cells compared to wild type C57BL/6J mice with retinal detachment (Sweigard et al., 2015). A similar result was observed in endothelial cell death in a model of ocular ischemia (Sweigard et al., 2014). Recent studies of mice deficient in key complement receptors (*C3aR*^−/−^ and *C5aR*^−/−^) have found that a- and b-wave signals from electroretinograms (ERGs) of these strains were impaired in mice that were 14 weeks, 6 months, and 12 months old compared to ERG readings from strain-matched mice that were 6 weeks old (Yu et al., 2012). Additionally, light microscopy in *C3aR*^−/−^ and *C5aR*^−/−^ mice revealed that inner nuclear layer (INL) and outer nuclear layer (ONL) thickness in 14-month-old mice were diminished compared to that of 3-month-old mice. These results suggest that C3aR and C5aR are necessary in maintaining normal retinal structure and function (Yu et al., 2012).

The complement system is active in the retina, RPE, and choroid under endogenous conditions. Immunohistochemistry has revealed that C1q is expressed in the retinal ganglion cell (RGC) layer even during the developmental stage (Stevens et al., 2007). Furthermore, Factor b (Collier et al., 2011), C3 (Anderson et al., 2010; Collier et al., 2011; Luo et al., 2011), and C5 (Copland et al., 2010) components have been detected in normal retinas. In mRNA analysis, a key component of the mannose-binding-lectin (Mbl) pathway was also identified in normal retinas (Luo et al., 2011). However, the role of these complement factors play under normal conditions is unclear. To fill this knowledge gap, this study focused on the effects of the three main complement pathways—classical, lectin, and alternative pathways—on retinal function and morphology during the normal aging process using complement-pathway deficient mice.

## Materials and methods

### Animals

The Massachusetts Eye and Ear Animal Care Committees approved all animal procedures and experiments prior to beginning, and all animals were treated in accordance with the guidelines set forth by the Association of Research for Vision and Ophthalmology.

In this study, C57BL/6 (stock no. 000664) mice were purchased from Jackson Laboratories (Bar Harbor ME, USA). *C3*^−/−^, *C5*^−/−^, *Fb*^−/−^, and *C1q*^−/−^ mice were a gift from J.D.L. at the University of Pennsylvania. *Mbl A/C*^−/−^ mice were a gift from G. Stahl at Brigham and Women's Hospital. All complement-deficient strains were in a C57BL/6 background. Each strain was maintained as a breeding colony in the Massachusetts Eye and Ear animal facility. Mice of all complement knockout stains utilized were screened for: RD1, RD2, RD3, RD6, RD7, RD8, and RD 10 mutations (TransnetYX®), and all strains were negative for these retinal degenerative genes.

### Electroretinography (ERG) recording

Full-field ERGs were recorded simultaneously from both eyes. Animals were dark-adapted overnight (>12 h). Mice were weighed and anesthetized with intraperitoneal injections of a mixture of ketamine (60 mg/kg) and xylazine (9 mg/kg). Mydriasis was achieved with one drop of 0.5% tropicamide with 5% phenylephrine. Corneal anesthesia was performed with a single drop of 0.5% proparacaine hydrochloride ophthalmic solution (Akorn Inc. Illinois). A warm heating pad was used to maintain body temperature (37°C).

ERGs were recorded using HMS's ERG LAB system (OcuScience, Nevada). Stimulus flashes were presented in a Ganzfeld bowl. Stimulus intensities ranging from −1.5 to 1.0 log cd s/m^2^ in 0.5-log units steps were used under dark-adapted conditions. Light stimuli were presented with a 1-min interval between successive stimuli.

### SD-OCT imaging

Before optical coherence tomography (OCT) imaging was performed, each animal was anesthetized by intraperitoneal Avertin injection (125 mg/kg), and the pupils were dilated with a drop of 0.5% tropicamide with 5% phenylephrine. Two repeated volumetric images, centered on the optic nerve head, were acquired in both eyes using spectral-domain OCT (SD-OCT; Bioptigen, Inc., Davis, NC). All SD-OCT images consisted of 1,000 A-scans and 100-averaged B scans (each B-scan was the average of three B-scans). These parameters correspond to the area of approximately 1.4 × 1.4 mm.

### SD-OCT image analysis

The RGC layer, inner plexiform layer and ganglion cell complex (IPL/GC), INL, and ONL were measured using SD-OCT B-scan cross-sectional images (Supplementary Figure 1A). To evaluate the cross-sectional thickness (in microns, μm) of each layer, manual calipers included in the Bioptigen software program were used. Retinal en-face scans were divided into inferior, nasal, temporal, and superior quadrants surrounding the central retinal subfield containing the optic nerve heads. One B-scan obtained approximately 400 μm from the optic nerve head (ONH) superiorly or inferiorly was selected for measurement of each retinal layer (Supplementary Figure 1B superior and inferior). Three B-scans that included the ONH or approximately 140 μm from the ONH superiorly and inferiorly were selected for measurement of each retinal layer in the nasal and temporal regions of the fundus. In the superior and inferior B-scan images, three measurement points were selected as follows: the central measurement point was determined as just superior or inferior of the ONH, and the other two points were defined at 300 μm nasally or temporally from this central measurement point. To measure the retinal thickness in the nasal or temporal area, three points were selected at 300 μm nasally or temporally from the ONH in the three B-scan images. Therefore, a total of 12 points were used to average the thickness of each retinal layer (Supplementary Figure 1B).

### Light microscopy and electron microscopy

Mouse eyes were enucleated and immersed in half strength Karnovsky's fixative (2% formaldehyde + 2.5% glutaraldehyde in 0.1 M sodium cacodylate buffer, pH 7.4; Electron Microscopy Sciences, Hatfield, Pennsylvania) at room temperature. An eyecup was created with each eye by dissecting away the anterior portion from the posterior portion. Eyecup samples were then placed back into half-strength Karnovsky's fixative for a minimum of 24 h under refrigeration. After fixation, samples were rinsed with 0.1 M sodium cacodylate buffer, post-fixed with 2% osmium tetroxide in 0.1 M sodium cacodylate buffer for 1.5 h, en bloc stained with 2% aqueous uranyl acetate for 30 min, then dehydrated with graded ethyl alcohol solutions, transitioned with propylene oxide, and resin infiltrated in tEPON-812 epoxy resin (Tousimis, Rockville, Maryland) utilizing an automated EMS Lynx 2 EM tissue processor (Electron Microscopy Sciences, Hatfield, Pennsylvania). The whole posterior eye cup was processed and embedded as a single block in transparent epoxy resin to prevent outer segment detachment during subsequent processing and sectioning procedures. The mid optic nerve head plane within the embedded eyecup was targeted for sectioning through the use of a stereoscope observing all directions of the transparent embedded block, then scored for sectioning using the optic nerve as an exterior reference. An initial precision saw cut was made into the embedded block away from the mid-level plane then ground down using a frosted slide. The edges of the eyecup tissue were trimmed using razor blades for ultramicrotomy. Sections were subsequently generated at 1 micrometer thickness and stained with toluidine blue stain. The orientation of photoreceptor outer segments was assessed in addition to the level through optic nerve head. The angle of cut and orientation in the both the x and y directions where made to generate a mid-level plane with regions of retinal photoreceptor outer segments oriented longitudinally prior to thin sectioning at 80 nanometers thickness for electron microscopy. Ultrathin sections (70–90 nm) were cut from the epoxy block using a Leica EM UC7 ultramicrotome (Leica Microsystems, Buffalo Grove, IL) and a diamond knife, collected onto 2 × 1 mm single slot formvar/carbon coated grids, and were stained with aqueous 25% Uranyl Acetate Replacement stain (Electron Microscopy Sciences, Hatfield, Pennsylvania) and Sato's lead citrate using a modified Hiraoka grid staining system. Grids were imaged using a FEI Tecnai G2 Spirit transmission electron microscope (TEM) (FEI, Hillsboro, Oregon) at 80 kV interfaced with an AMT XR41 digital CCD camera (Advanced Microscopy Techniques, Woburn, Massachusetts) for digital TIFF file image acquisition. TEM imaging of all layers of the retina was used to capture representative regions. When analyzing the number of dense inclusions in the outer plexiform layer (OPL), 10 images taken at x11,000 magnification were selected every 50 μm from the optic nerve head in each mouse strain.

### Statistical analysis

All data are expressed as means ± SE. ERG comparisons in a-wave or b-wave signals were made between 6-week-old and 6-month-old mice using two-way analysis of variance (ANOVA), and comparisons of retinal thickness were analyzed using a two-tailed student's *t*-test. The Pearson *r*-test was used to estimate the correlation between retinal thickness and ERG amplitude. *P* < 0.05 were considered statistically significant.

## Results

### Reduced retinal function with age in complement knockout mice

**Figure 1:**
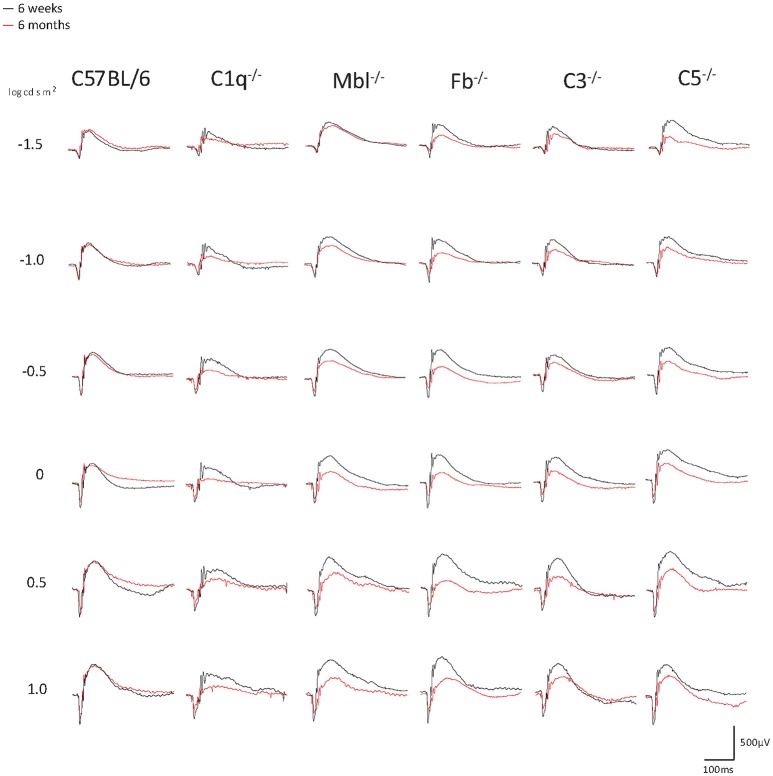
Representative examples of ERG a- and b-wave responses in young (6 weeks) and adult (6 months) mice in each experimental group. In C57BL/6 mice, the amplitudes of a- and b-waves between young and adult mice were almost identical. On the other hand, both amplitudes were impaired in aged *C1q*^−/−^, *Mbl*^−/−^, *Fb*^−/−^, *C3*^−/−^, and *C5*^−/−^ mice. A scale was shown at the bottom right.

**Figure 2:**
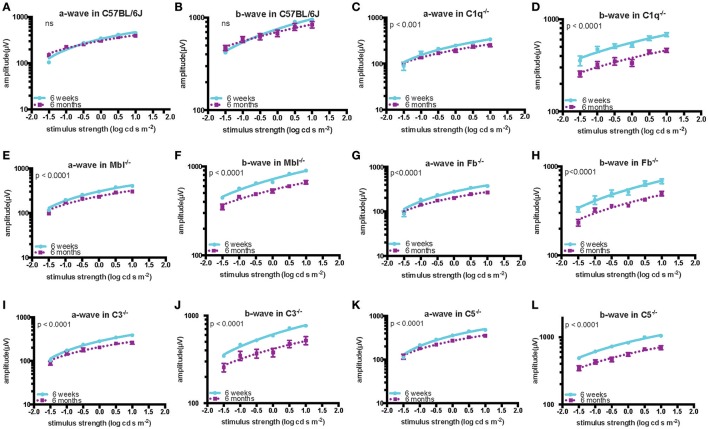
Quantification of ERG amplitudes in a- and b-waves of young (6 weeks) and adult (6 months) mice in each experimental group. In C57BL/6 mice **(A,B)**, there was no significant difference in the amplitude of a- and b-waves between the young (*n* = 5) and old (*n* = 5) mice. By contrast, both amplitudes were significantly decreased in old compared to in young *C1q*^−/−^ mice (a-wave: *p* < 0.001, b-wave: *p* < 0.0001; young *n* = 3, adult *n* = 3), *Mbl*^−/−^ mice (a-wave: *p* < 0.0001, b-wave: *p* < 0.0001; young *n* = 6, adult *n* = 5), *Fb*^−/−^ mice (a-wave: *p* < 0.001, b-wave: *p* < 0.0001; young *n* = 4, adult *n* = 8), *C3*^−/−^ mice (a-wave: *p* < 0.001, b-wave: *p* < 0.0001; young *n* = 6, adult *n* = 4), and *C5*^−/−^ mice (a-wave: *p* < 0.001, b-wave: *p* < 0.0001; young *n* = 3, adult *n* = 3) **(C–L)**.

**Figure 3:**
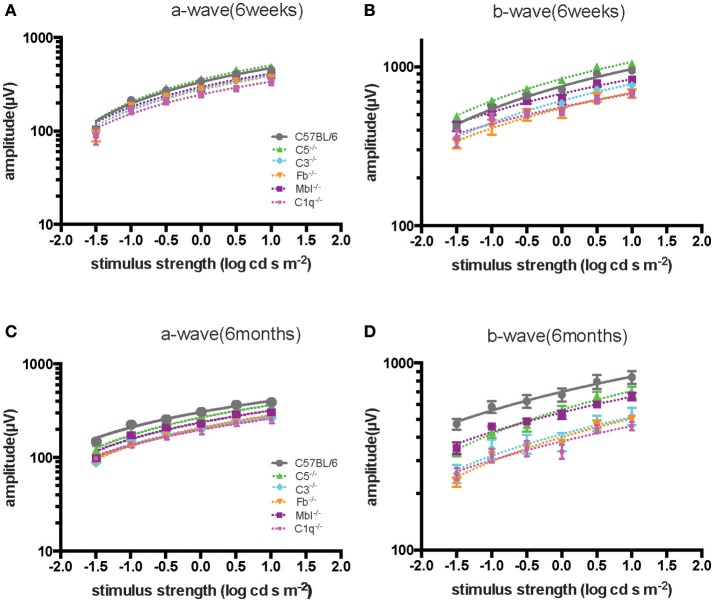
Comparison of ERG a- and b-wave amplitudes among experimental mouse strains at 6 weeks **(A,B)** and 6 months **(C,D)** of age. The amplitude of a- and b-waves in complement knockout strains in *C1q*^−/−^, *Mbl*^−/−^, *Fb*^−/−^, and *C3*^−/−^ mice were lower than those of C57BL/6 mice at each age, except for *C5*^−/−^ mice at 6 weeks of age.

Significant decreases in the ERG amplitude of both a-wave and b-waves were detected in all strains of complement knockout mice that were 6 months old, compared to that of complement knockout mice (strain-matched mice) at 6 weeks of age; however, no such age-related differences were noted in C57BL/6 mice (Figures 1, 2). The extent of signal loss was more severe in the classical pathway knockout (*C1q*^−/−^) and alternative pathway knockout (*Fb*^−/−^) strains (Figure 3).

### Thinning of retinal layers with age in complement knockout mice

**Figure 4:**
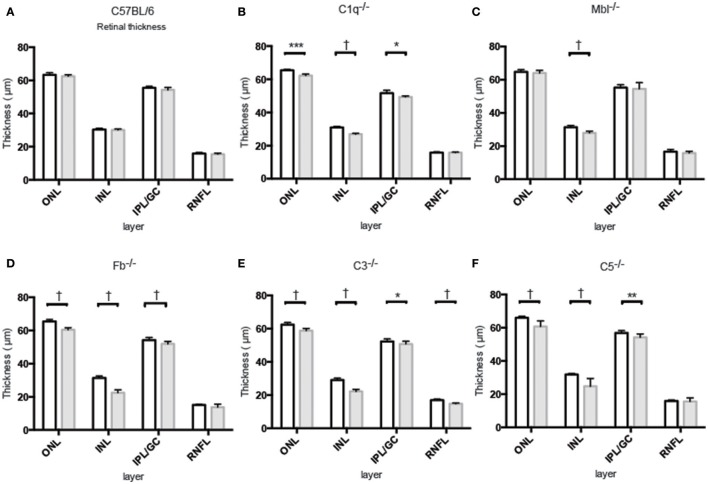
Mean thickness of retinal sub-layers measured by Spectral Domain Optical Coherence Tomography in young (6 weeks) and adult (6 months) mice in each experimental group. **(A)** There was no significant thinning in any retinal layer between 6-week-old and 6-month-old C57BL/6 mice. **(B–F)** Contrastingly, INL thinning was identified in all complement knockout strains at the age of 6 months. Values and statistical analysis are summarized in Table 1. RNFL, retinal nerve fiber layer; IPL/GC, inner plexiform layer/ganglion cell; INL, inner nuclear layer; ONL, outer nuclear layer. Bar = 100 μm. ^*^p < 0.05, ^**^p < 0.01, ^***^p < 0.001, ^†^*p* < 0.0001.

Inner nuclear layer (INL) thickness was significantly decreased in *C1q*^−/−^, *Mbl*^−/−^, *Fb*^−/−^, *C3*^−/−^, and *C5*^−/−^ mice at 6 months of age compared to that in strain-matched mice at 6 weeks of age; however, no such age-related differences in INL thickness were found between 6-week-old and 6-month-old C57BL/6 mice (Figure 4). Specifically, we observed the following reductions in INL thickness in 6-month-old mice compared to 6-week-old mice: 23% reduction in *C1q*^−/−^, 21% in *Mbl*^−/−^, 36% in *Fb*^−/−^, 34% in *C3*^−/−^, and 23% in *C5*^−/−^ mice. There were significant decreases in the thickness of the IPL/GC in C57BL/6, *C1q*^−/−^, *Fb*^−/−^, *C3*^−/−^, and *C5*^−/−^ mice; however, only a 1.3–4.7% reduction was identified (Table 1).

**Table 1:** Changes in thickness of different retinal layers in 6-week-old or 6-month-old mice from each experimental strain.

**Layer**	**Strain**	**Thickness (SE)** μ**m**				***P*-values**
		**6 weeks**	***n***	**6 months**	***n***	
**ONL**						
	C57BL/6	63.35(0.38)	10	62.53(0.38)	6	0.18
	*C1q^−/−^*	65.46(0.22)	4	62.25(0.36)	6	<**0001**
	*Mbl^−/−^*	64.73(0.51)	7	64.03(0.51)	10	0.34
	*Fb^−/−^*	65.50(0.48)	5	61.03(0.32)	14	<**0.0001**
	*C3^−/−^*	62.45(0.34)	14	58.83(0.46)	8	<**0.0001**
	*C5^−/−^*	65.94(0.40)	6	60.76(0.55)	7	<**0.0001**
**INL**						
	C57BL/6	30.38(0.24)	10	30.07(0.27)	6	0.42
	*C1q^−/−^*	30.90(0.34)	4	26.88(0.22)	6	<**0.0001**
	*Mbl^−/−^*	31.34(0.42)	7	27.88(0.34)	10	<**0.0001**
	*Fb^−/−^*	31.43(0.48)	5	23.32(0.46)	14	<**0.0001**
	*C3^−/−^*	29.03(0.31)	14	22.14(0.46)	8	<**0.0001**
	*C5^−/−^*	31.78(0.30)	6	24.68(0.80)	7	<**0.0001**
**IPL/GC**						
	C57BL/6	55.47(0.31)	10	54.22(0.61)	6	0.06
	*C1q^−/−^*	51.67(0.84)	4	49.39(0.24)	6	<**0.05**
	*Mbl^−/−^*	55.22(0.64)	7	54.53(1.17)	10	0.64
	*Fb^−/−^*	54.25(0.68)	5	52.32(0.41)	14	<**0.01**
	*C3^−/−^*	52.32(0.40)	14	50.79(0.57)	8	<**0.05**
	*C5^−/−^*	56.93(0.56)	6	54.24(0.34)	7	<**0.01**
**RNFL**						
	C57BL/6	15.93(0.20)	10	15.51(0.26)	6	0.22
	*C1q^−/−^*	15.75(0.23)	4	15.76(0.15)	6	0.98
	*Mbl^−/−^*	16.72(0.44)	7	15.83(0.31)	10	0.11
	*Fb^−/−^*	15.08(0.14)	5	15.15(0.48)	14	0.86
	*C3^−/−^*	16.91(0.17)	14	14.77(0.16)	8	<**0.0001**
	*C5^−/−^*	15.90(0.25)	6	15.54(0.36)	7	0.70

*RNFL, Retinal nerve fiber layer; IPL/GC, inner plexiform layer/ganglion cell; INL, inner nuclear layer; ONL, outer nuclear layer. Significant differences in each layer between young and old animals for each KO strain were detected and noted in bold*.

### Positive correlation between the amplitude of b-waves and the thickness of the INL and IPL/GC

According to retinal single cell recordings, it is thought that the ERG is a mass retinal response in which the a-wave is generated by photoreceptors and the b-wave primarily reflects activation of bipolar cells. Thus, we analyzed the correlation between a-wave amplitude and ONL thickness, the correlation between b-wave amplitude and INL thickness, and the correlation between b-wave amplitude and IPL/GC thickness. A positive correlation between the b-wave amplitude and INL thickness (*r*^2^ = 0.5263, *p* < 0.01), as well as a correlation between b-wave amplitude and IPL/GC thickness (*r*^2^ = 0.7608, *p* < 0.001), were noted. However, no significant correlation was identified between the a-wave amplitude and ONL thickness (Supplementary Figures 2, 3).

### Histologic analysis

**Figure 5:**
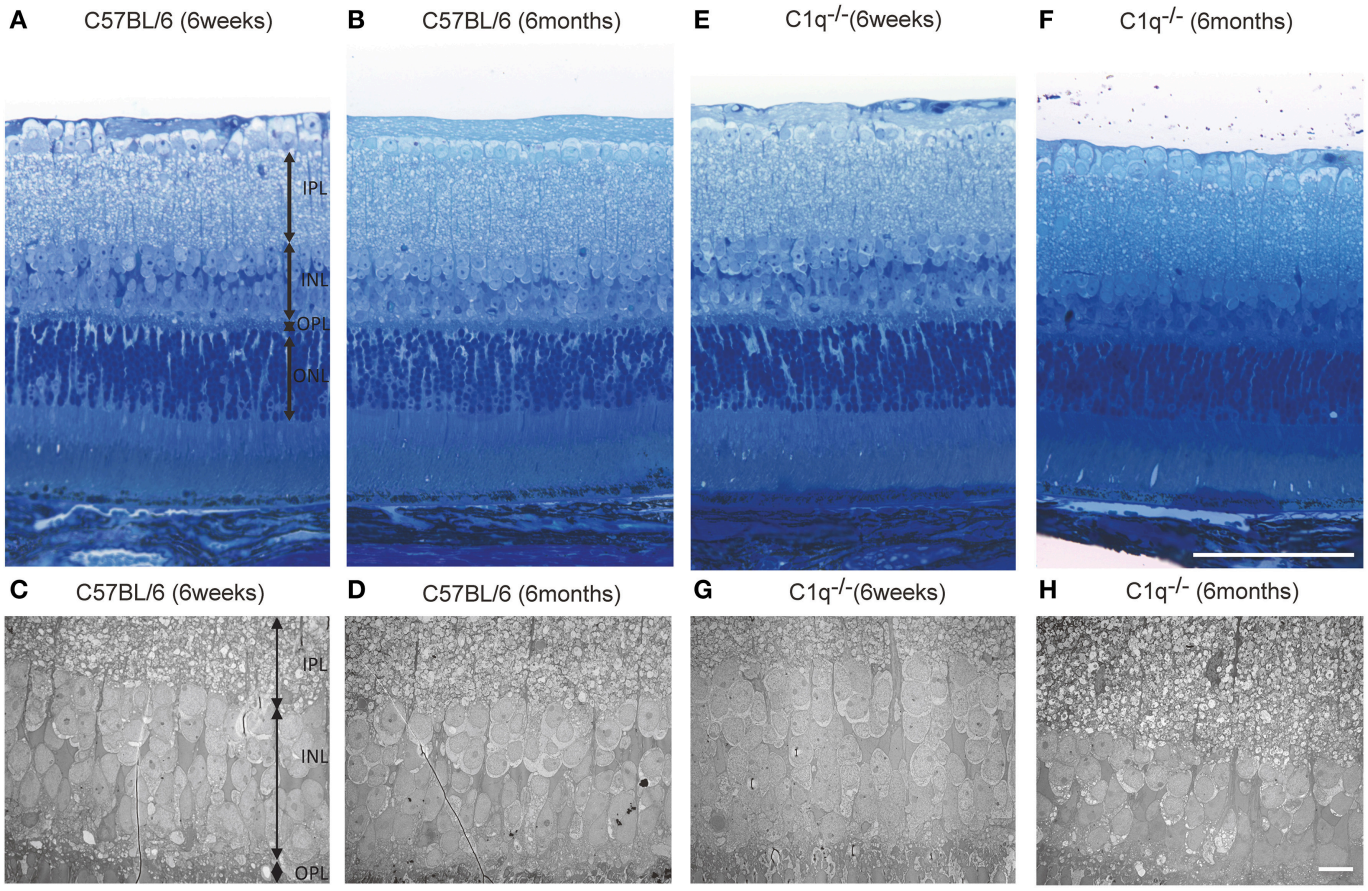
Representative light microscopic and electron microscopic images comparing the retinal layer thickness of C57BL/6 **(A–D)** and *C1q*^−/−^ **(E–H)** mice at 6 weeks **(A,C,E,G)** and 6 months **(B,D,F,H)** of age. **(A–D)** No thinning in any retinal layer were observed between 6-week-old and 6-month-old C57BL/6 mice. **(E–H)** In *C1q*^−/−^ mice at 6 months of age, the thickness of inner nuclear layer seemed to be thinner than that in *C1q*^−/−^ mice at 6 weeks of age. Bar = 100 μm, **(F)** Bar = 10 μm **(H)**.

**Figure 6:**
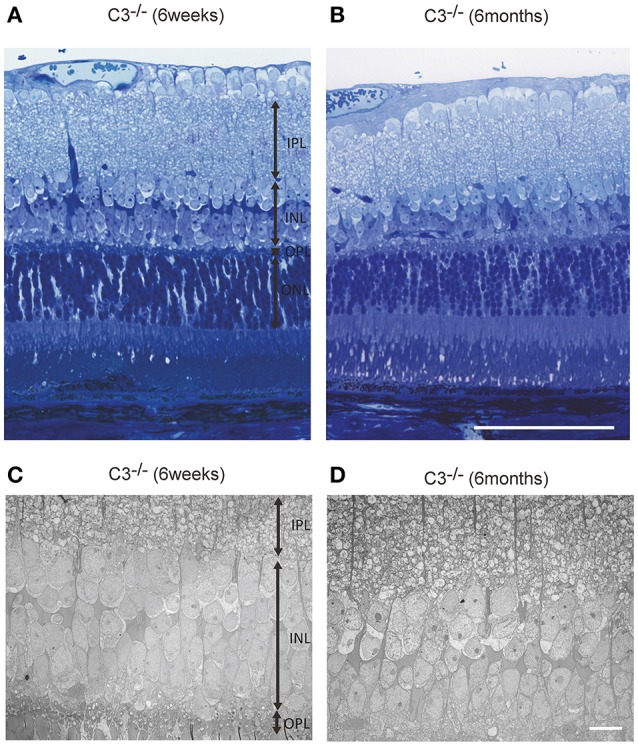
Representative light microscopic **(A,B)** and electron microscopic images **(C,D)** comparing the retinal layer thickness of *C3*^−/−^ mice at 6 weeks **(A,C)** and 6 months of age **(B,D)**. **(A–D)** The thickness of the inner nuclear layer in *C3*^−/−^ mice at 6 months of age **(B,D)** appears thinner than that in *C3*^−/−^ mice at 6 weeks of age **(A,C)**. **(A,B)** Bar = 100 μm. **(C,D)** Bar = 10 μm.

**Figure 7:**
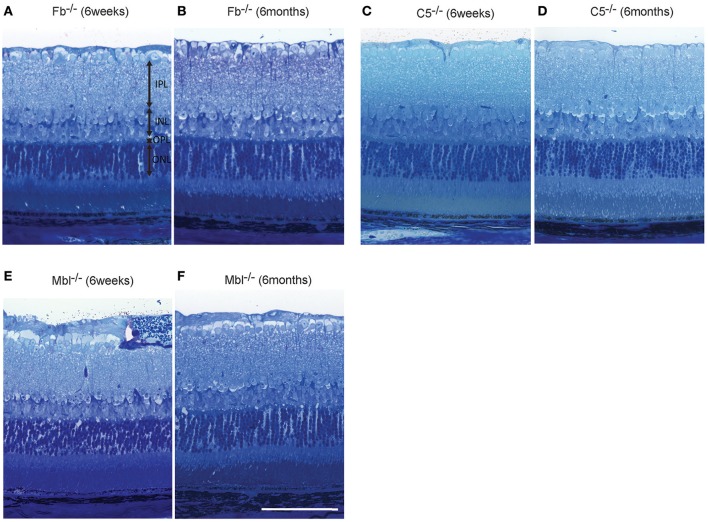
Representative comparison of retinal layer thickness between 6-week-old and 6-month-old *Fb*^−/−^ **(A,B)**, *C5*^−/−^ **(C,D)**, and *Mbl*^−/−^ **(E,F)** mice. **(A–F)** There was no apparent difference in retinal thickness of each layer between mice at 6 weeks of age and those at 6 months of age. **(A–F)** Bar = 100 μm.

**Figure 8:**
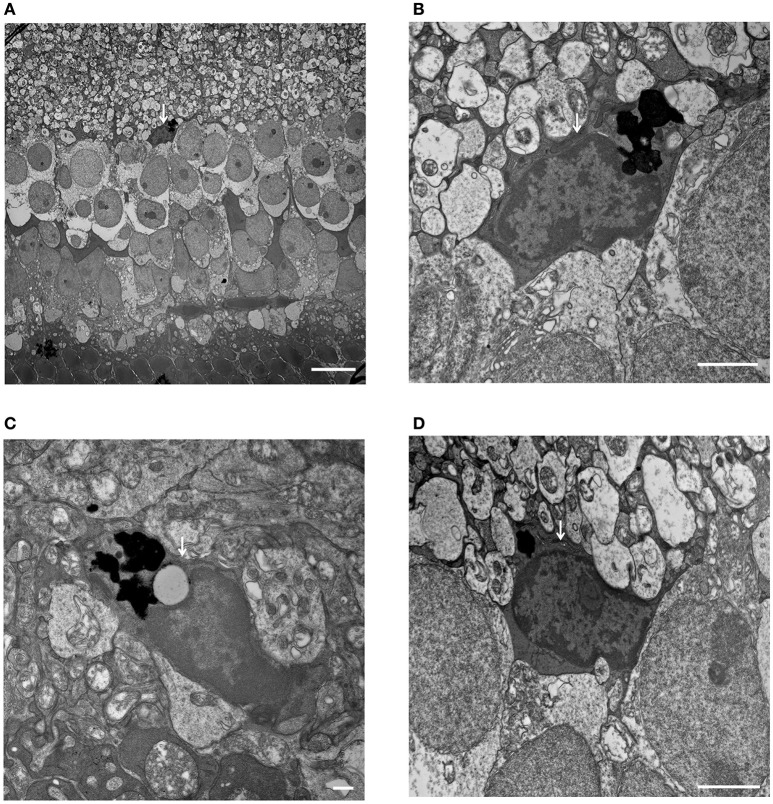
Phagocytized microglial cells in the inner nuclear layer of *C1q*^−/−^ mice at 6 months of age. (**A–D**, white arrows) **(A)** A microglial cell phagocytizing dead cells, with dense particles observed in the microglial cell body at the border between the inner nuclear layer and inner plexiform layer (magnification: x1,400). **(B)** High magnification image of **(A)** (magnification: x9,300). **(C,D)** Microglial cells containing dense particles were detected in other parts of the inner nuclear layer. (**C**: x13,000, **D**: x9,300). **(A)** Bar = 10 μm, **(B)** Bar = 2 μm, **(C)** Bar = 500 nm, **(D)** Bar = 2 μm.

**Figure 9:**
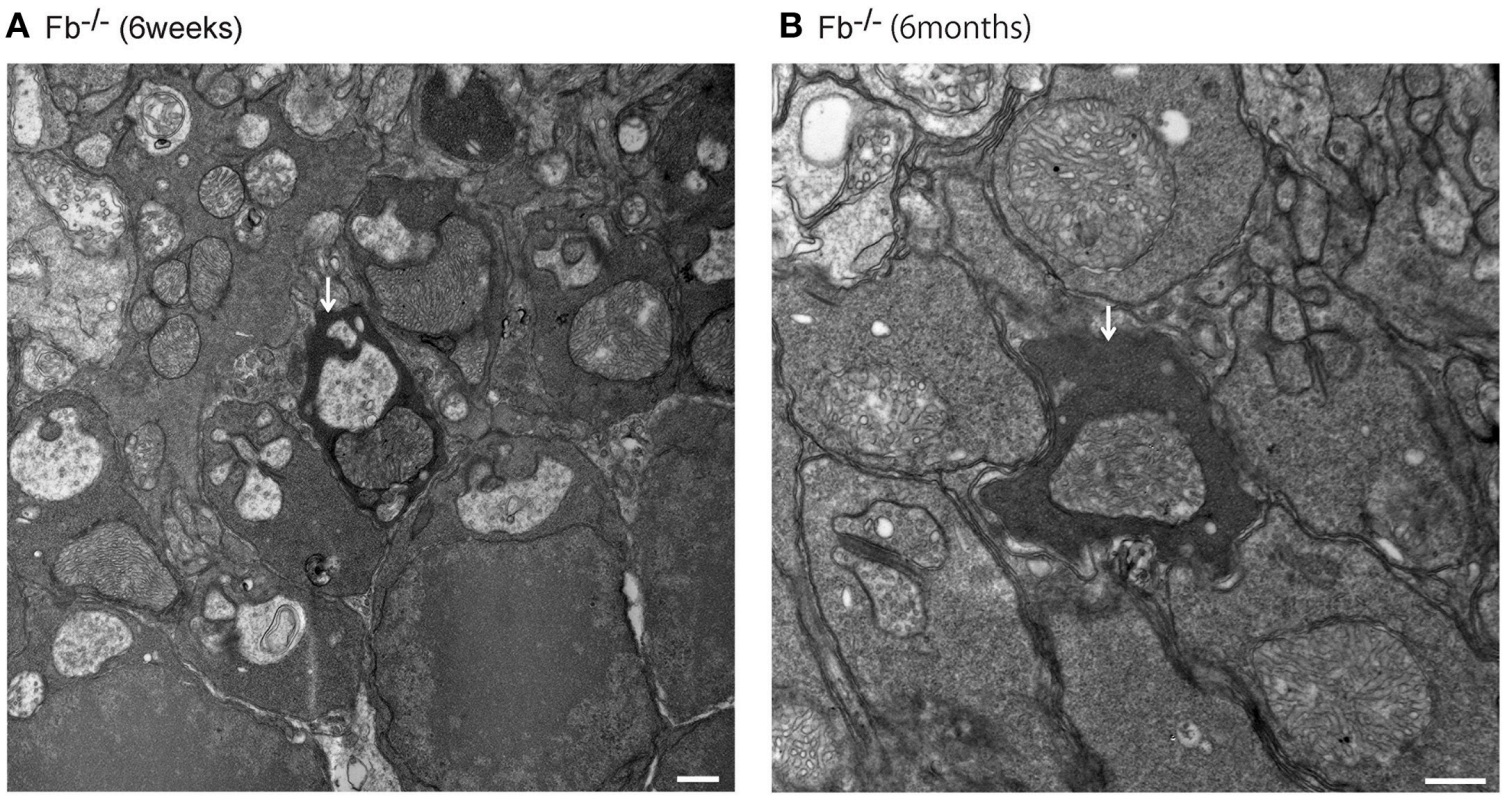
Assessment of dense inclusions in the outer plexiform layer (OPL) (**A,B**, white arrows). Dense inclusions in the OPL have been reported previously in microglia-depleted retinas, and were due the destruction of pre-synaptic termini in the OPL (Wang et al., 2016). The presence of dense inclusions was identified in alternative complement pathway deficient mice and corresponded to the border between the tip of photoreceptors and neighboring bipolar cells. **(A)** *Fb*^−/−^ mice at 6 weeks of age. Bar = 500 nm. **(B)** *Fb*^−/−^ mice at 6 months of age. Bar = 500 nm.

Light microscopic and electron microscopic images were obtained in each mouse strain in order to define the morphological changes induced in the retina when there was a deficiency in complement system activity. INL thinning was observed via electron and light microscopy in *C1q*^−/−^ and *C3*^−/−^ mice that were 6 months of age relative to mice at 6 weeks of age (Figures 5, 6). In *Fb*^−/−^, *C5*^−/−^, and *Mbl*^−/−^ mice, there was no difference between 6-week-old and 6-month-old mice (Figure 7). In particular, phagocytized cells were identified in the INL in the *C1q*^−/−^ strain (Figure 8). Dense inclusions in the OPL have previously been identified as the degeneration of the junction between photoreceptors and bipolar cells (Wang et al., 2016). Our retinal samples also contained dense inclusions in the OPL in *Mbl*^−/−^, *Fb*^−/−^, *C3*^−/−^, and *C5*^−/−^ mice that were 6 months old. Furthermore, similar dense inclusions were identified in strain-matched mice that were 6 weeks old (Figure 9, Supplementary Figure 4, Table 2).

**Table 2:** The number of dense inclusions in each mouse strain at 6-weeks or 6-months of age.

**Strains**	**Age**	
	**Young (6 weeks)**	**Old (6 months)**
C57BL/6J	0	0
*Fb^−/−^*	3	5
*C3^−/−^*	16	9
*C5^−/−^*	5	11
*C1q^−/−^*	0	0
*Mbl^−/−^*	2	3

## Discussion

Retinal functional abnormalities were detected in *C1q*^−/−^, *Mbl*^−/−^, *Fb*^−/−^, *C3*^−/−^, and *C5*^−/−^ mice at 6 months of age compared to in mice at 6 weeks of age under normal, disease-free conditions. This functional deterioration seemed to correlate with thinning of the inner retina in these strains. Additionally, dense inclusions in the OPL were observed via electron microscopy in *Mbl*^−/−^, *Fb*^−/−^, *C3*^−/−^, and *C5*^−/−^ mice at 6 months of age. These structural changes in the OPL may potentially contribute to retinal functional abnormalities, at least in some complement knockout mice.

In C57BL/6 mice, a mild decrease in a-wave and b-wave amplitudes was observed in 6-month-old mice compared to in 6-week-old mice; however, the changes were not significant in this study, indicating that retinal function is preserved from 6 weeks to 6 months in C57BL/6 mice. A previous study detected significant reductions in a-wave and b-wave amplitudes in 12-month-old mice, but such reductions were limited in 6-month-old mice, as compared to 2-month-old mice in the C57BL/6 strain (Li et al., 2001). In another study, authors compared ERGs from 1-, 4-, and 17-month-old mice, showing significantly decreased ERG amplitudes in 17-month-old mice compared to in 1-month-old mice; however, no major differences in ERG amplitude were detected at least in b-waves between 1-month-old and 4-month-old mice in same strain (Gresh et al., 2003). These trends are quite similar to those found in our study.

Decreased amplitudes of both a- and b-waves at 6 months of age were identified not only in alternative pathway knockout mice (*Fb*^−/−^, *C3*^−/−^, and *C5*^−/−^), but also in classical (*C1q*^−/−^) as well as mannose-binding-lectin pathway (*Mbl*^−/−^) knockout mice. A previous study in 2012 explored how deficiencies in alternative pathway components such as C3, C3aR, and C5aR impacted ERG amplitude in mice (Yu et al., 2012). The authors identified decreased b-wave amplitudes in *C3*^−/−^ mice at 12 months of age, as well as decreased a- and b-wave amplitudes in *C3aR*^−/−^ and *C3aR*^−/−^*C5aR*^−/−^ mice at 14 weeks, 6 months, and 12 months of age. In addition, thinning of the INL and ONL was observed by 14 months of age in C3aR-deficient mouse strains, as analyzed by retinal histological sections (Yu et al., 2012). These results seem to support our ERG and OCT data in alternative pathway-deficient mice (*Fb*^−/−^, *C3*^−/−^, and *C5*^−/−^).

In this study, we discovered that INL thickness in SD-OCT was significantly decreased in *C1q*^−/−^, *Mbl*^−/−^, *Fb*^−/−^, *C3*^−/−^, and *C5*^−/−^ mice at 6 months of age, compared to strain-matched mice, at 6 weeks of age and that this significantly correlated with b-wave amplitude. In addition, light microscopy revealed thinning of the INL in *C3*^−/−^ and *C1q*^−/−^ mice in our study. Specifically, we found that thinning in the INL inner region was more severe than that of the INL outer region. We therefore postulate that thinning of inner retinal layer at 6 months of age may be a key contributor to the deteriorating retinal function observed in these strains. However, there were some discrepancies in the results of INL thickness between SD-OCT and light microscopic finding among KO lines (Figures 4–7, Table 1). Thinning of the INL observed by SD-OCT in *C3*^−/−^ and *C1q*^−/−^ corresponded to thinning observed by light microscopic analysis, although there were some discrepancies in results of INL thickness between SD-OCT and light microscopic findings in other strains. We regarded thickness in OCT (Figure 4) as more accurate than thickness in HE or EM (Figures 5–7), due to the possibility that fixation can modify the thickness of tissues.

The mechanism for neuronal loss in the INL and ONL in complement knockout strains, remains unclear. Yu et al. demonstrated that decreased PKCα staining in the INL correlated with a reduction in the number of Calbundin D28 positive cells, and found swelling of the outer segment in aged *C3aR*^−/−^ and *C3*^−/−^ mice in comparison to wild type mice (Yu et al., 2012). Hoh and colleagues revealed that *C3*^−/−^ mice had significant photoreceptor loss and thickening of the Bruch's membrane as well as reduced amplitudes in photopic and scotopic responses in 12-month-old mice, as compared to C57BL/6 mice of the same age (Hoh Kam et al., 2013). These results suggested that deficiency of complement components might cause impairment of signaling function in the inner and outer retinal layers.

Hoh therefore concluded that inhibition of C3 for treatment of age-related macular degeneration (AMD) patients might be deleterious (Hoh Kam et al., 2013). Our ERG findings in *C3*^−/−^ mice are in accordance with this study, and strengthen the possibility that lack of C3 in the retina might contribute to reduced retinal function over time.

Interestingly, we detected dense inclusions in the OPL via electron microscopy, which may correspond to the degeneration of synapses between photoreceptors and bipolar cells (Somogyi et al., 1977; Linder et al., 1987; Wang et al., 2016), in complement pathway-deficient (*Fb*^−/−^, *Mbl*^−/−^, *C3*^−/−^, and *C5*^−/−^) mice at 6 months of age. Dense inclusions in the OPL have been reported previously in microglia-depleted retinas, with diminished scotopic and photopic responses due to the destruction of pre-synaptic termini in the OPL (Wang et al., 2016). These structural changes in the OPL and electrophysiological changes in b-wave amplitude in microglia-depleted retinas were similar to our results in normal aging eyes (Wang et al., 2016). Based on our findings, we hypothesize that the complement system may maintain retinal integrity under normal homeostatic conditions. Nevertheless, it is still unclear why complement depletion may lead to degeneration in the retina. Dense inclusions were observed in OPL, but their number was limited (Figure 9, Table 2, Supplementary Figure 4). We evaluated the number of dense inclusions around the disc area (and not centrally) as we sought to evaluate the number of dense inclusions at the same position in all of our complement-deficient mice. Thus, this could be why the number of dense inclusions was limited.

When analyzing morphological changes in INL and IPL by electron microscopy, we focused on morphological changes in amacrine cells, bipolar cells and horizontal cells. We recognized the star-like shape of nuclei in amacrine cells as an index. The Kolmer's organelle is highly important in order to identify horizontal cells (Hogan et al., 1971; Richard et al., 2001). However, there were no obvious changes in these cells in both young and old complement-deficient strains. Next, we focused on synaptic ribbons between photoreceptors and bipolar cells, and compared the number of synaptic ribbons between young and old mice in complement-deficient strains. Still, there were no changes in the number of synapse ribbons in all complement-deficient strains (Supplementary Figure 5). In addition, although we tried to identify bipolar cells synapses by focusing on the numerous number of synaptic vesicles in axons of bipolar cells, it was difficult to identify and compare them among complement-deficient strains in a quantitative analysis. Moreover, we did not observe any distinct morphological changes in Müller cells in any of our complement-deficient strains. In C1q strains, we identified microglial cells at the inner surface of INL, which appeared to include phagocytosed cells. From the aspect of the location of these phagocytosing cells, it is possible that microglial cells had engulfed bipolar or amacrine cells in at least the C1q strain (Figure 8).

Of note, our study has shown the functional (Figure 2) and morphological changes (Figure 7D, Supplementary Figures 6, 7) in *C5*^−/−^ mice at 6 months of age. In addition, there appeared to be swelling and folding of the outer segment in C5^−/−^ mice at 6 months of age (Figure 7D, Supplementary Figures 6, 7). By contrast, previous studies did not show significant reductions in ERG amplitude and retinal thickness in *C5aR*^−/−^ mice with increasing age (Yu et al., 2012). We suspect this may be due to C5 signaling being transmitted through two receptors (Ward, 2009, 2010; Bosmann et al., 2013; Li et al., 2013), such as C5aR1 and C5L2, which is suggestive that severe dysfunction only in the absence of both receptors.

Recently, evidence has been accumulating regarding the association between genetic alterations in complement factor H (CFH) with the development of age-related macular degeneration (AMD) in clinical studies. CFH has also been a topic of intense investigation in basic research (Anderson et al., 2010; Kondo et al., 2011). Photoreceptor loss and decreased amplitude in ERG scotopic responses were observed in 12-month-old CFH knockout mice (*Cfh*^−/−^) (Ding et al., 2015). Additionally, the study noted an apparent decrease in INL thickness. Outer retinal degeneration resembling retinal changes seen in AMD was observed in 10-month-old mice lacking CD46, which is thought to regulate the aggregation of C3b and C4 (Lyzogubov et al., 2016). Basal laminar deposits were observed in Fb^−/−^ mice at the age of 6 months in our study (Supplementary Figure 8). These results suggest that a lack of alternative pathway components or an imbalanced complement system could result in retinal degeneration in younger animals, even under normal conditions. Although a deficiency in CFH or CD46 could potentially enhance alternative pathway activity and cause retinal degeneration observed with AMD, it is also possible that the lack of a fully functional complement system could equally contribute to alteration of normal retinal homeostasis in the context of aging.

To the best of our knowledge, this is the first study to suggest that, in addition to C3, C3aR, and C5aR(Yu et al., 2012), Factor b, C5, C1q, and Mbl A/C of the complement system are involved in the maintenance of retinal integrity under normal aging conditions. Further studies are needed to detail the association between the complement system and other immune components endogenous conditions, which would enhance our understanding of the mechanisms of age-related retinal diseases.

## Author contributions

Drafting the article and conception or design of the work: RM and KC. Critical revision of the article, data analysis and interpretation: RM, YO, CK, DH, JL, and KC.

### Conflict of interest statement

The authors declare that the research was conducted in the absence of any commercial or financial relationships that could be construed as a potential conflict of interest.

## Abbreviations

ERG: Electroretinogram
EM: electron microscopy
INL: inner nuclear layer
RD: retinal degeneration
SD-OCT: spectral domain optical coherence tomography
IPL/GC: inner plexiform layer/ganglion cell layer
ONL: outer nuclear layer
ONH: optic nerve head.
