# Supplementary figures and images for: The Complement System Is Critical in Maintaining Retinal Integrity during Aging
Source: Front Aging Neurosci. 2018 Feb 15;10:15. doi: 10.3389/fnagi.2018.00015 (PMC5818470; doi:10.3389/fnagi.2018.00015)

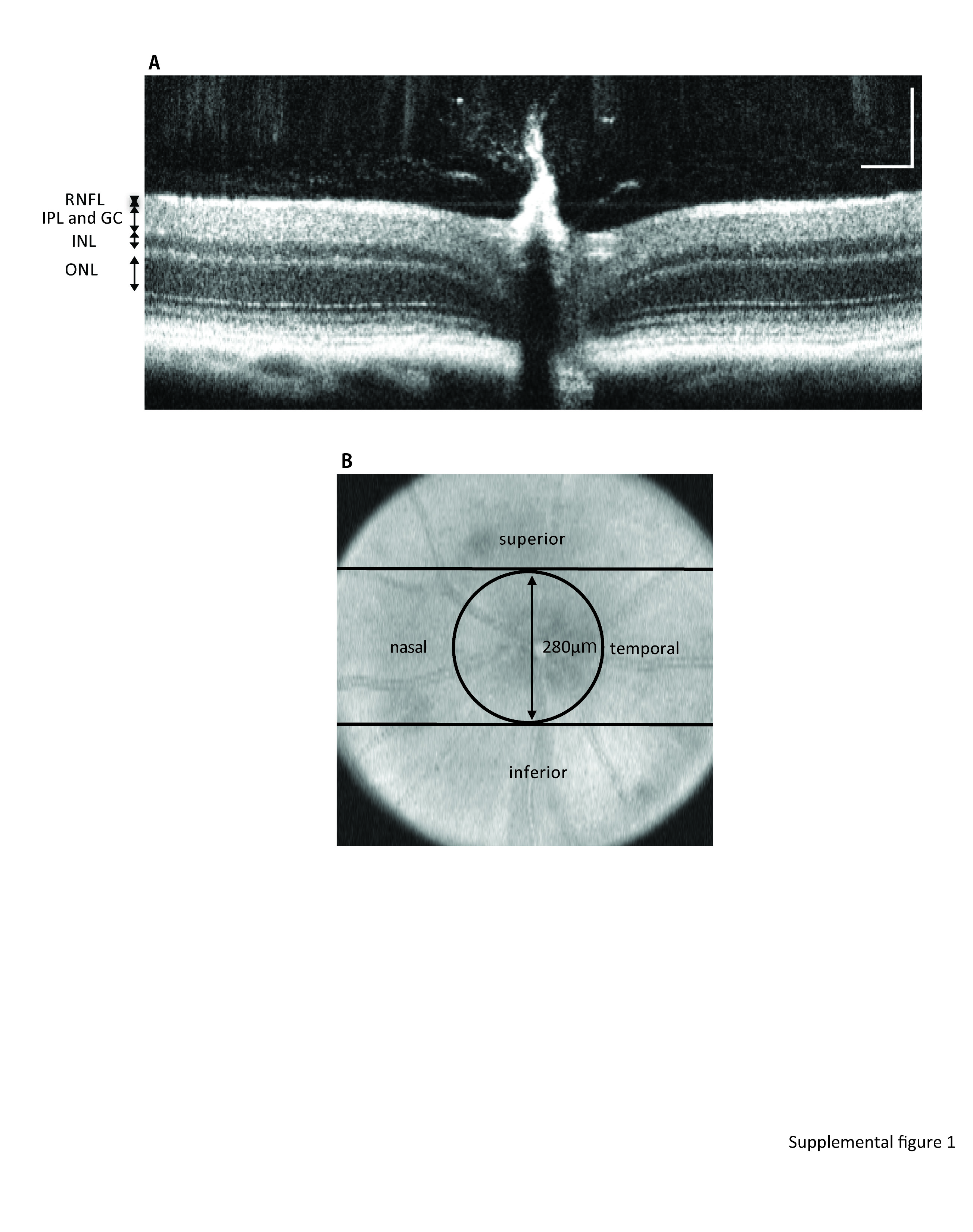

Supplement: Supplementary file 1 [file Image1.jpg]

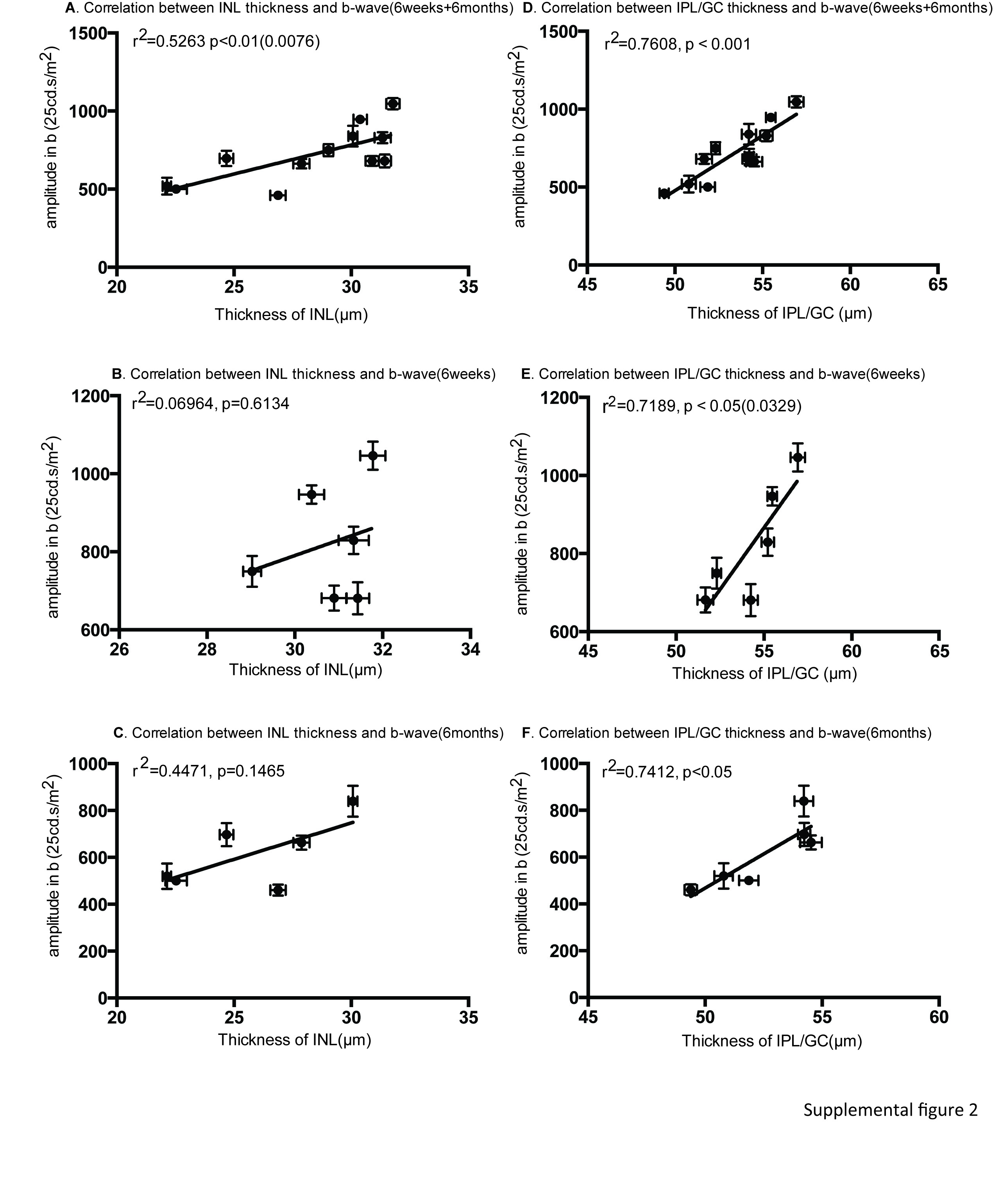

Supplement: Supplementary file 2 [file Image2.jpg]

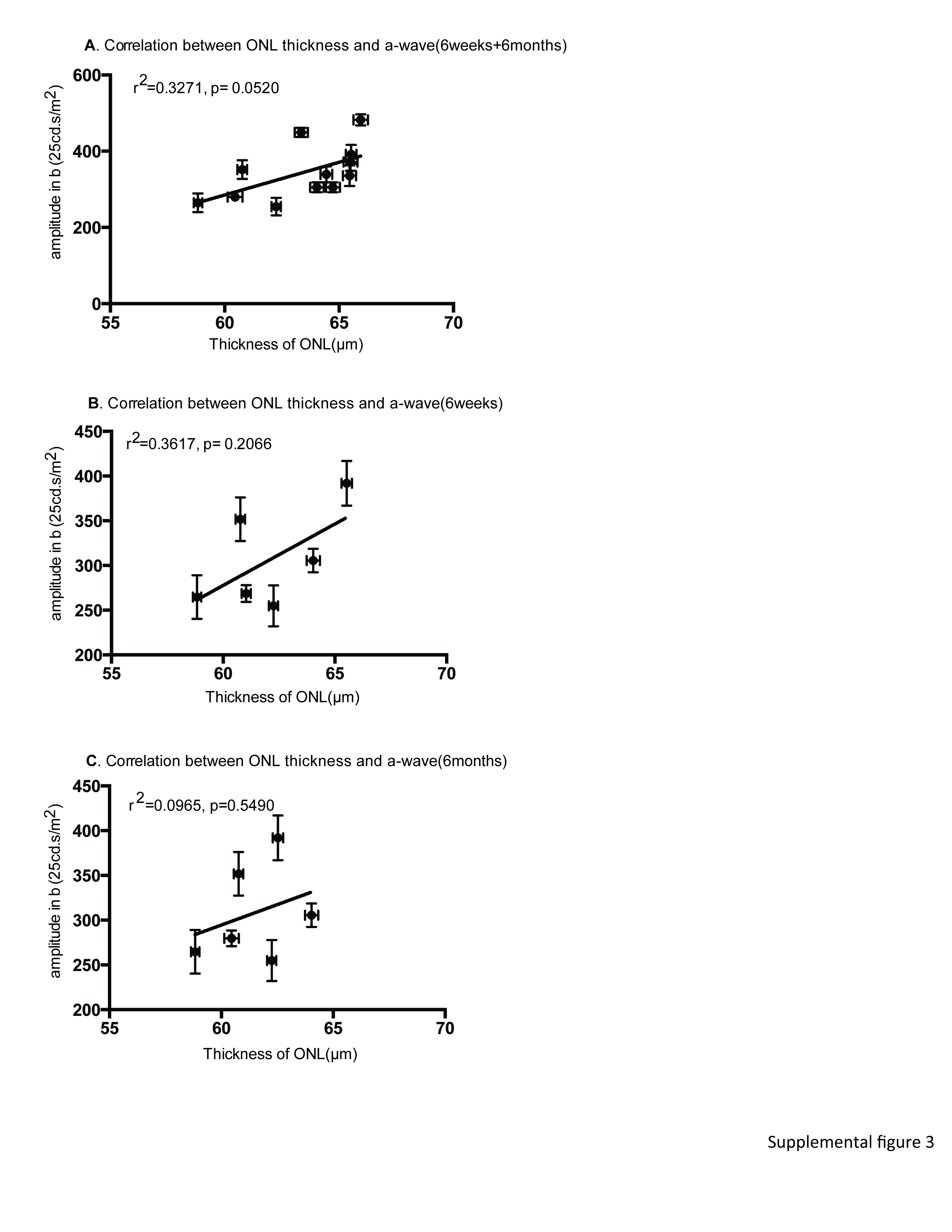

Supplement: Supplementary file 3 [file Image3.jpg]

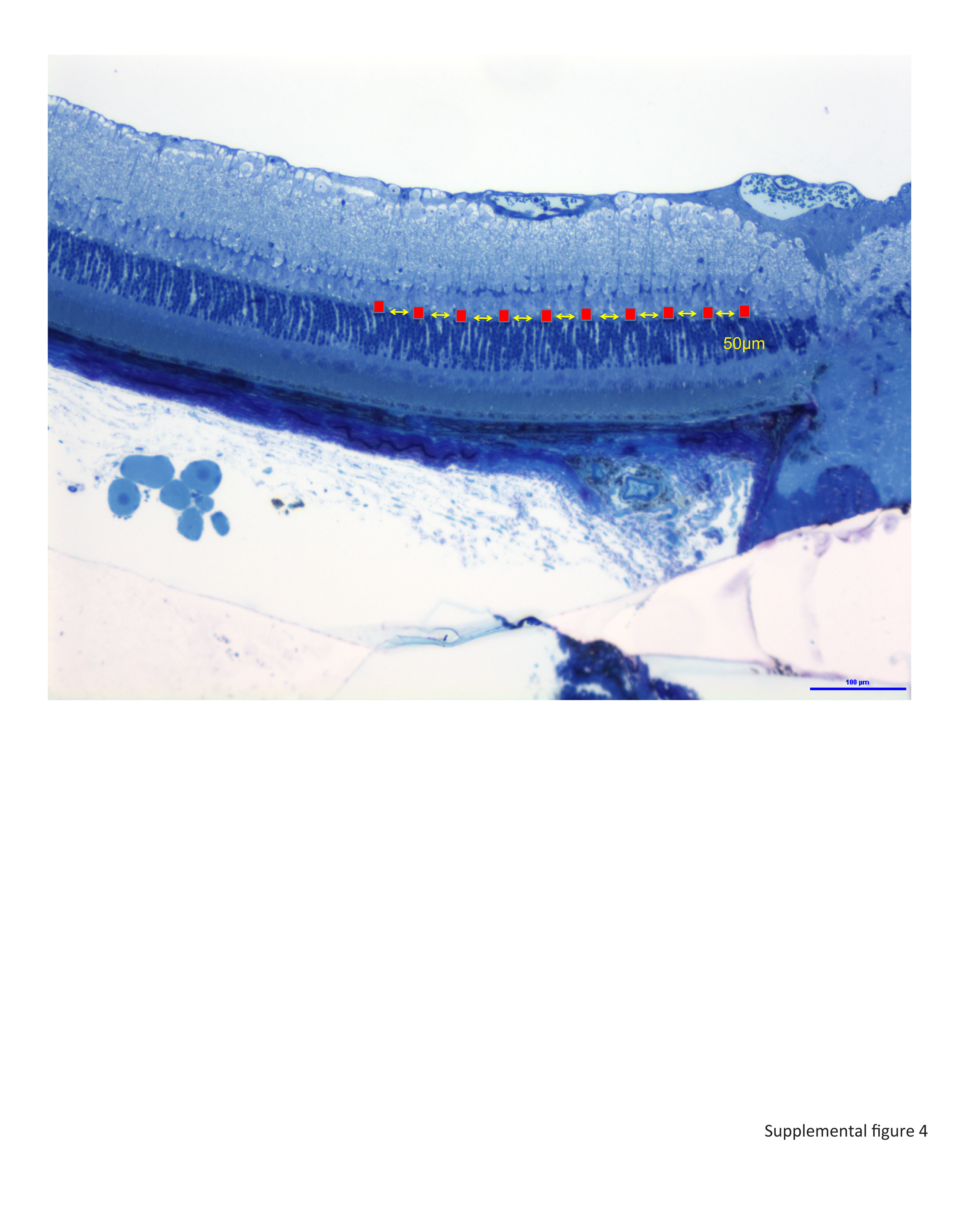

Supplement: Supplementary file 4 [file Image4.jpg]

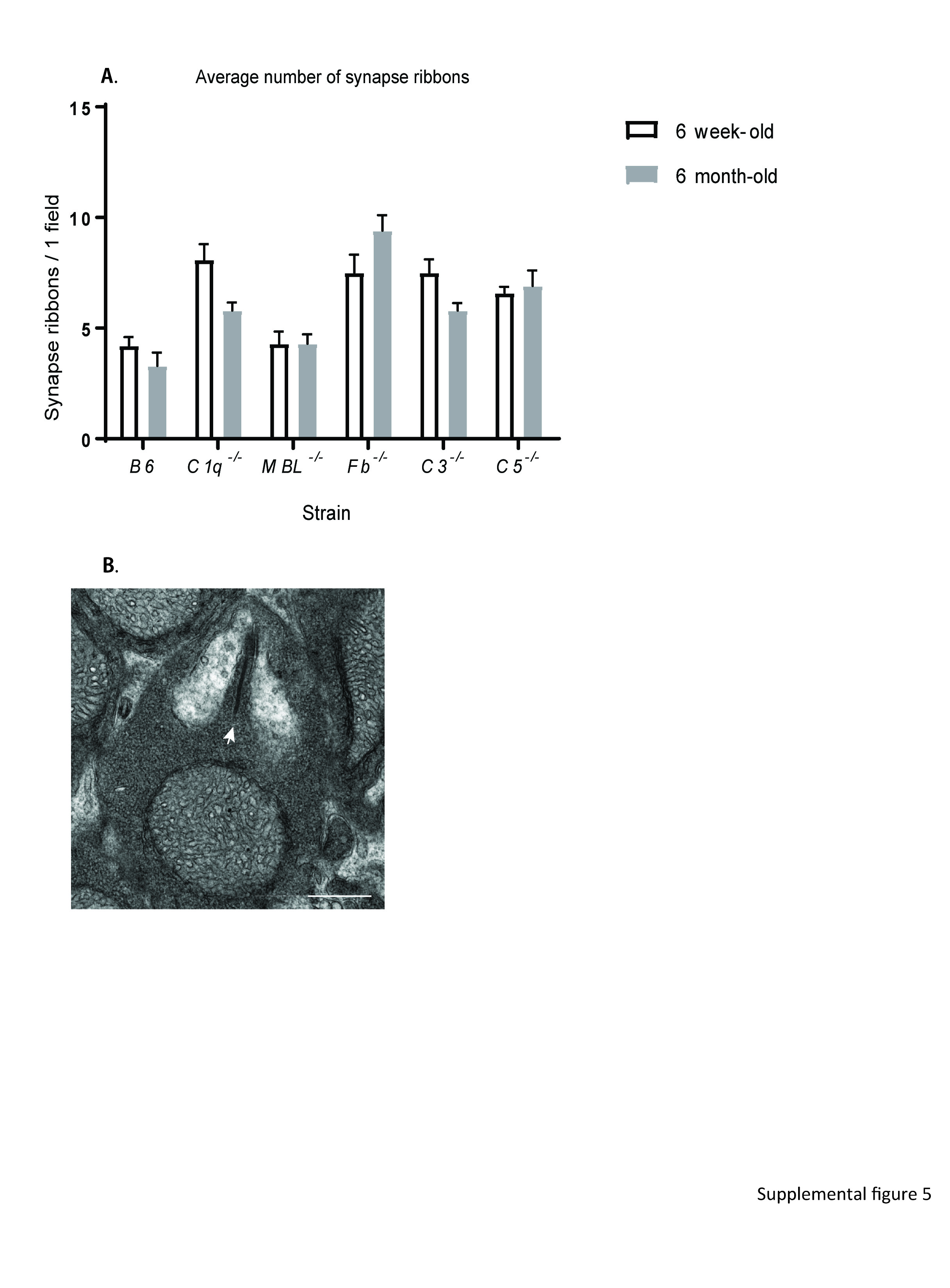

Supplement: Supplementary file 5 [file Image5.jpg]

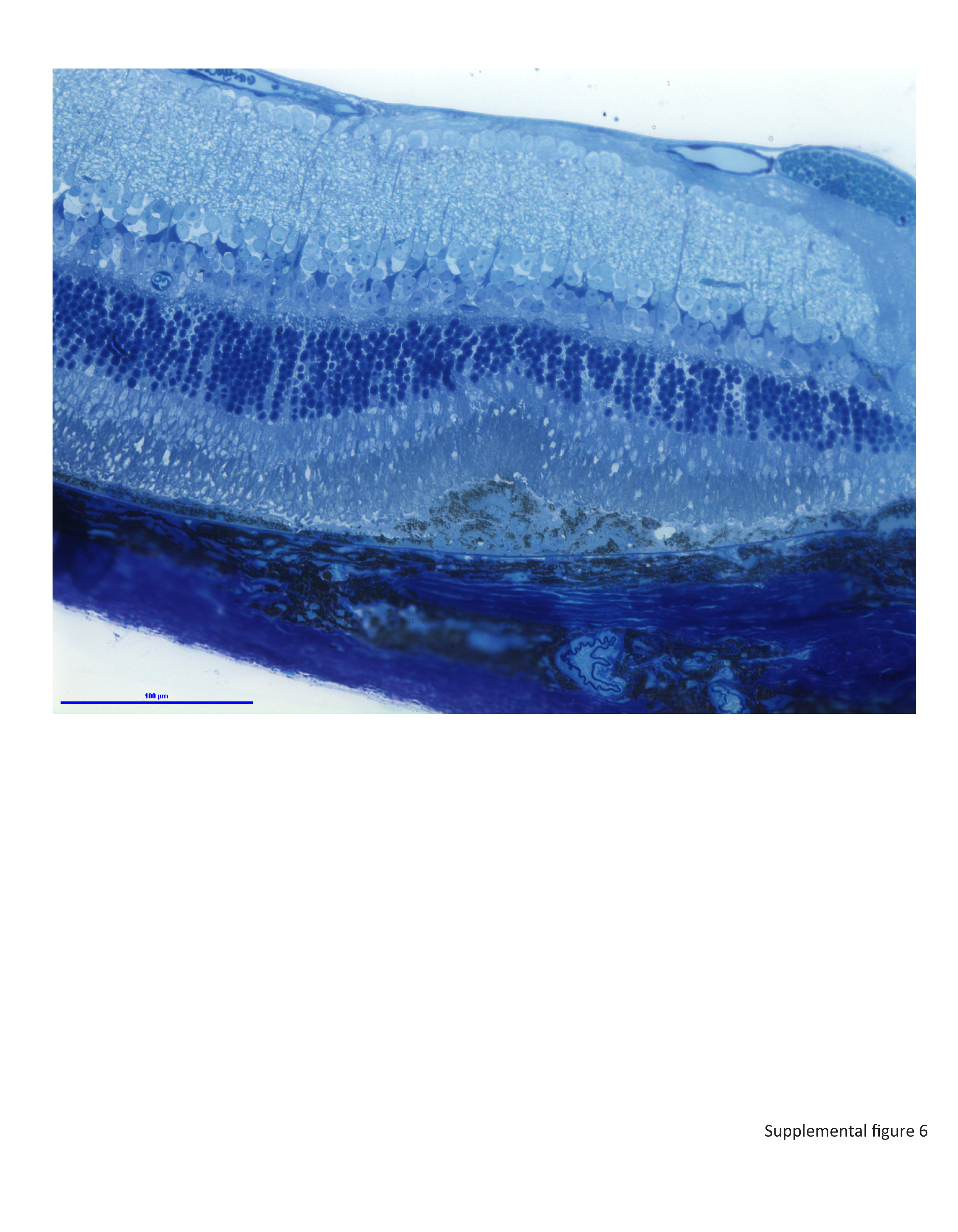

Supplement: Supplementary file 6 [file Image6.jpg]

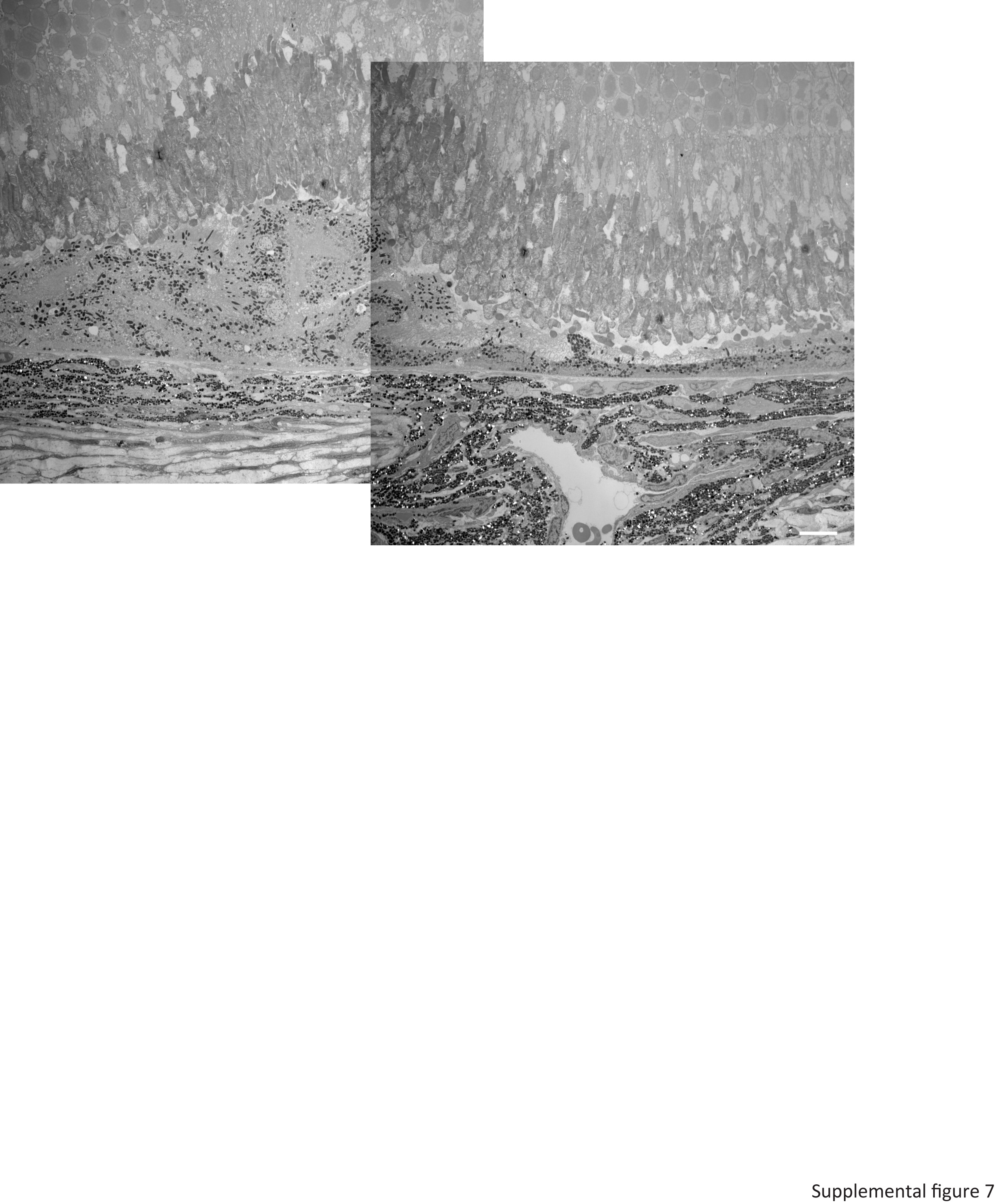

Supplement: Supplementary file 7 [file Image7.jpg]

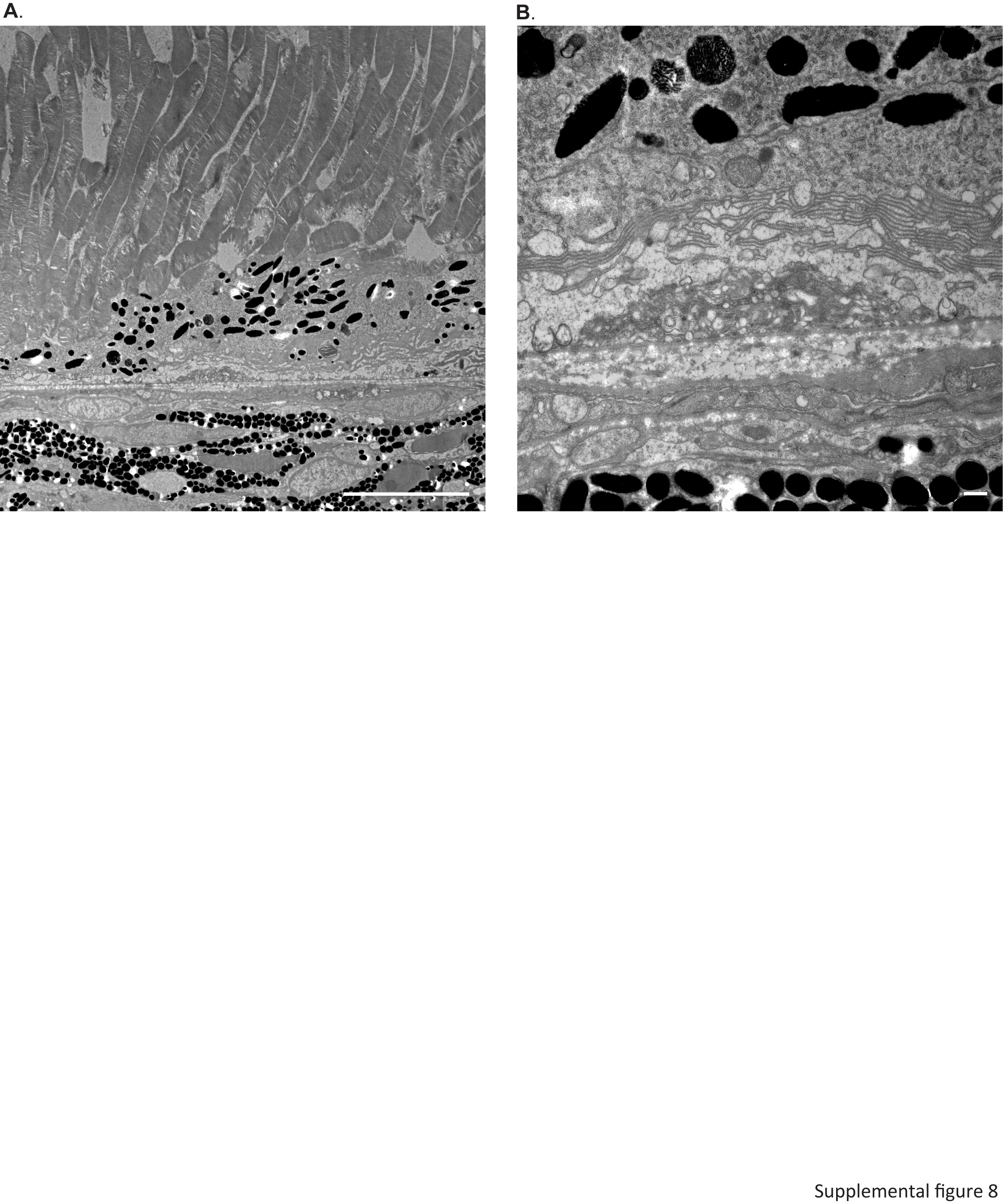

Supplement: Supplementary file 8 [file Image8.jpg]
