# Supplementary material for: The Complement System Is Critical in Maintaining Retinal Integrity during Aging
Source: Front Aging Neurosci. 2018 Feb 15;10:15. doi: 10.3389/fnagi.2018.00015 (PMC5818470; doi:10.3389/fnagi.2018.00015)
Supplement: Supplementary file 9 [file DataSheet1.docx]

Supplementary Material

**The complement system is critical in maintaining retinal integrity during aging**

Ryo Mukai, Yoko Okunuki, Deeba Husain, Clifford B. Kim, John D. Lambris, and

Kip M. Connor*

*Correspondence: Kip M Connor: [kip_connor@meei.harvard.edu](mailto:kip_connor@meei.harvard.edu)

Supplemental figures legend

**Supplemental Figure 1**.

Retinal scans and posterior segmental layer thickness analysis. **A**. B-scan image of a mouse retina at the level of the optic nerve, segmentation is illustrated. RNFL: retinal nerve fiber layer, IPL/GC: inner plexiform layer/ ganglion cell, INL: inner nuclear layer, ONL: outer nuclear layer. Scale Bar = 100 μm. **B**. En-face view of the posterior segment centered on the optic nerve head. The retinal area was divided into superior, inferior, nasal, and temporal regions as shown. Three points were selected in each region to calculate the average thickness of each retinal layer.

**Supplemental figure 2**.

Single regression analysis of the thickness of retinal layers (INL:**A-C**; IPL/GC: **D-F**) and the amplitude of b-waves in all experimental eyes of all strains from 6-week-old and 6-month-old mice. A strong correlation between the IPL/GC and the b-wave amplitude was observed (**D-F**). Additionally, a significant correlation was detected between INL thickness and b-wave amplitude (**A**).

**Supplemental figure 3**.

Single regression analysis of the thickness of retinal layers (ONL: **A-C**) and the amplitude of a-waves in all experimental eyes of all strains from 6-week-old and 6-month-old mice. Not significant but positive correlation between the thickness of the ONL and the a-wave amplitude was observed.

**Supplemental figure 4**.

The strategy for analyzing the number of dense inclusions and in the outer plexiform layer.

Ten images (red rectangles) taken at x11,000 magnification was obtained every 50 μm from the optic nerve head, and the number of dense inclusions was counted in each image.

Quantified data is shown in Table 2. Scale Bar = 100 nm.

**Supplemental figure 5**.

**A**. Average number of synapse ribbons between photoreceptors and bipolar cells at the age of 6 weeks or 6 months for each complemental knock out strain and wild-type (C57BL/6) mice. Ten electron microscopic images of x11000 in the synapsed were obtained every 50 μm from optic disc to distal position of the retina, and the number of synapse ribbons was counted in each image. **B**. A representative synapse ribbon between a photoreceptor and a bipolar cell (white arrow). Scale Bar = 500 nm.

**Supplemental figure 6**.

Subretinal mass and retinal pigment epithelial proliferation were observed at the age of 6 months in the *C5^-/-^* mouse strain. Scale Bar = 100 μm.

**Supplemental figure 7**.

Electron microscopic images of the subretinal mass and retinal pigment epithelial proliferation seen in Supplemental figure 4 at the age of 6 months in the *C5^-/-^* mouse strain. Magnification x890, Scale Bar = 10 μm.

**Supplemental figure 8**.

Basal laminar deposit observed in Fb-/- mice at the age of 6 months using x2900 magnification (**A**: Scale Bar = 10 μm) and x13000 (**B**: Scale Bar = 500 nm) by electron microscopy.
